# Influence of Infill Pattern on Mechanical Behavior of Polymeric and Composites Specimens Manufactured Using Fused Filament Fabrication Technology

**DOI:** 10.3390/polym13172934

**Published:** 2021-08-31

**Authors:** María Jesús Martín, Juan Antonio Auñón, Francisco Martín

**Affiliations:** 1 Department of Civil, Materials, and Manufacturing Engineering, University of Malaga, C/Dr. Ortiz Ramos s/n, 29071 Málaga, Spain; mjmartin@uma.es; 2 Department of Mechanical, Thermal, and Fluids Engineering, University of Malaga, C/Dr. Ortiz Ramos s/n, 29071 Málaga, Spain; jaaunon@uma.es

**Keywords:** fused filament fabrication, polylactic acid, acrylonitrile butadiene styrene, nylon, glass fiber, carbon fiber, infill orientation, tensile behavior

## Abstract

This paper presents the results of a comparative evaluation of the tensile strength behaviors of parts obtained by additive manufacturing using fused filament fabrication (FFF) technology. The study investigated the influences of the deposition printing parameters for both polymers and fiber-reinforced polymers. Polymeric materials that are widely used in FFF were selected, including acrylonitrile butadiene styrene (ABS), polylactic acid (PLA), and nylon. Carbon and glass continuous fibers were used to reinforce the nylon matrix in composite materials. The study utilized two manufacturing methods. Polymers were manufactured using an Ultimaker 2 Extended+ device and the fiber-reinforced polymer specimens were obtained using a Markforged Mark Two printer. The entire set of specimens was eventually subjected to destructive monoaxial tensile tests to measure their responses. The main goal of this study was to estimate the effect of the different infill patterns applied (zig-zag, concentric, and four different orientations lines) on the mechanical properties of pure thermoplastic materials and reinforced polymers. Results show a spectacular increase in the tensile stress at break, which for polymers reaches an average value of 27.53 MPa compared to 94.51 MPa in the case of composites (increase of 70.87%). A similar increase occurs in the case of tensile stress at yield with values of 31.87 MPa and 105.98 MPa, respectively, which represents an increase of 69.93%. The influence of the infill of the fiber is decisive, reaching, in the 0-0 arrangement, mean values of 220.18 MPa for tensile stress at break and 198.26 MPa for tensile stress at yield.

## 1. Introduction

Basically, additive manufacturing refers to technologies that create three-dimensional objects by laying down material, layer upon layer, in a precise shape. Nowadays, the variety of different processes based on this principle makes additive manufacturing one of the leading sectors, and the number of technologies developed has dramatically increased in the last decade, with innovations such as the inclusion of metallic materials in large parts using different processes in Yilmaz et al. [1], conducting process comparisons in [2,3], Rosen [4] develops design guides for additive manufacturing, and Popescu et al. [5] offers a study on parameters of the most extended process (FFF). One of the most well-known additive manufacturing processes is material extrusion, where spooled polymers are extruded through a heated nozzle moving horizontally (x-y axes) while the bed moves vertically (z axis), allowing a part to be manufactured by laying down layer upon layer of the melted material [2]. These processes are studied by means of different approaches like the applications of neural network in the deposition paths [6], thermal stability [7], configuration parameters [8,9], increase of the pressure in the deposition of the material using roller pressure [10], measurement of the in-plane (XY) temperature field on the build plate [11], or difference in toolpath planning [12].

The vast selection of raw materials, which include polymers like PLA or ABS [13,14,15,16], ceramics, metals, composites [17,18], and even food items, along with the relatively low cost of the equipment and versatility of the parameter combinations [13,14,15,16,17,18], suggest that additive manufacturing technologies have development potential in a wide range of diverse fields, including the aerospace, manufacturing, medical prototyping [19,20] and biomedical in Sodupe-Ortega et al. [21], and material characterization fields.

**Figure 1:**
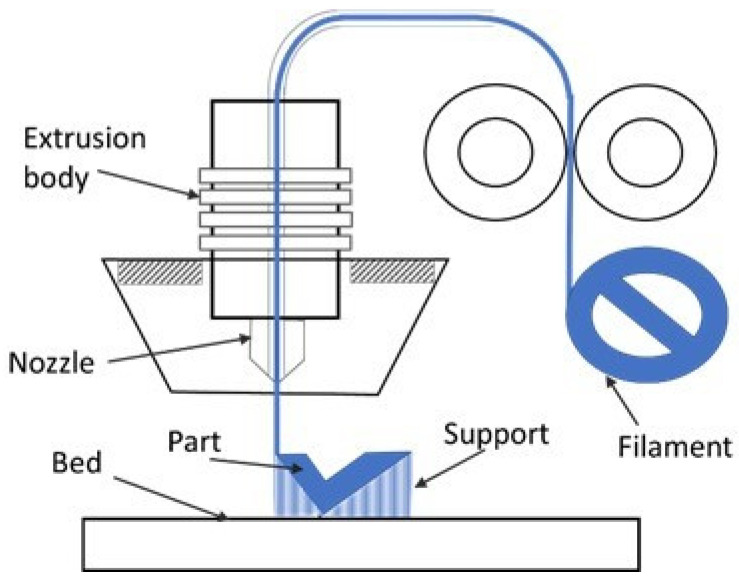
Schematic of fused filament fabrication (FFF) process.

Among the additive manufacturing techniques based on the melt extrusion method, the use of fused filament fabrication (FFF) processes (Figure 1) [5,6,7,8,9,10,22,23] has become increasingly widespread, and they currently have considerable development potential. These processes create parts by extruding a melted material, generally in the form of filaments, through nozzles with different diameters [24].

FFF technology is presented as one of the most widespread due to its low manufacturing cost, the versatility of materials that can be used, and the flexibility it presents in terms of manufacturing parameters (limited in some manufacturers), but it also presents limitations, and perhaps the main one is the fact that parts made by FFF show an anisotropic behavior [25].

In the Fused Filament Fabrication, basic and support materials processes can be extruded, depending on the 3D printer equipment, using either the same nozzle, different ones [26], or present particular cases as Baumann and Scholz [27] in which the fiber is deposited above the partially fabricated specimen. In the case of the fabrication of composite parts [2,26,27,28,29,30], usually it is necessary to use two independent nozzles, one for the matrix material and the other for the reinforcement fiber.

Therefore, to characterize the mechanical behavior of an FFF part, it is essential to understand the effects that the FFF parameters have on the anisotropic material properties. Parameters such as the layer height, nozzle diameter, raster angle [31,32] or infill pattern [33,34] of the melted filaments [35], for both the pure polymer and fiber in a composite material, and infill density must be taken into account in the mechanical response of the specimen obtained using this process [5,7,8,22,36,37].

In this project, the influences of the main FFF printing parameters were studied by the characterization of different materials subjected to destructive uniaxial tensile tests [7,8,38,39,40,41,42,43,44,45]. Even though there is still not an abundant number of publications referring to these emerging technologies, with regard to specific processes using the FFF technique, a large number of studies have been conducted to analyze the behaviors of specimens using different criteria, highlighting the porosity [46,47], dimensional accuracy [48,49,50], influence of temperature [11] and the rheological behavior [51], coating and impregnation of reinforcement fibers [52,53,54], part distortion [55,56], Domingo-Espin et al. on fatigue behavior [57,58], and bond formation [59,60].

Thus, a wide range of geometrical deposition patterns has been considered for pure polymers, such as nylon [2], polylactic acid (PLA) [22,26,48,61,62,63,64], and acrylonitrile butadiene styrene (ABS) [7,8,36,56,57,65], and composite materials (nylon matrices with carbon or glass fiber reinforcement) [66,67].

From an overall point of view, the design principles of specimens, along with the velocities and trajectories involved in the process, have been investigated [68,69,70,71]. As an emergent technology, there have been many studies on and innovations for these processes [10,60,72,73]. Such roller pressure [10], interlaminar bonding performance [60], five-axis 3D printing machine [72], or using automation [73].

## 2. Materials and Methods

### 2.1. Materials

The main objective of this project was to analyze how the mechanical properties of different materials were influenced by the application of different deposition patterns in the FFF processes. The materials studied were polymers and polymers with fiber as fillers. A large number of studies have been conducted to make comparative evaluations of PLA and ABS (Ultimaker, Utrecht, The Netherlands), which are the most commonly used polymers [74]. These materials have similar Young’s modulus or yield strength [75] values but differ in their physical and chemical characteristics. ABS is highly resistant to abrasion or impact, while PLA has low cost and withstands a temperature increase. Therefore, it is an excellent option for additive manufacturing. Additionally, this work also included nylon (Ultimaker, Utrecht, The Netherlands), which is a material with significantly different elongation and mechanical resistance properties compared to the previously mentioned materials (up to 40 times greater). Nylon was used as the matrix for the polymer reinforced with carbon or glass fiber (Table 1).

This project used a double investigative approach. On one hand, an attempt was made to establish the impact of the inherent conditions for this technology. In this type of process, the raw material is deposited onto a platform to construct a part layer by layer with different orientations, causing a characteristic anisotropic behavior and a potential weakness due to the low adherence between layers. On the other hand, the study considered the modification of the mechanical performance of a printed continuous fiber-reinforced polymer, consisting of a nylon matrix with carbon or glass fibers as filler, Li, C. et al. [58,76,77]. In this case, the infill pattern of the fiber was also a major determinant factor in the mechanical response of the specimens.

### 2.2. 3D Printer Equipment

**Figure 2:**
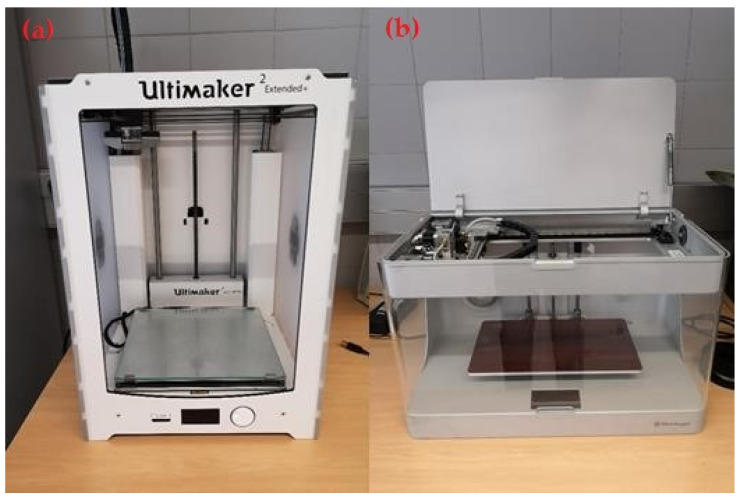
(**a**) Ultimaker 2 Extended+ printer; (**b**) Markforged Mark Two printer.

**Figure 3:**
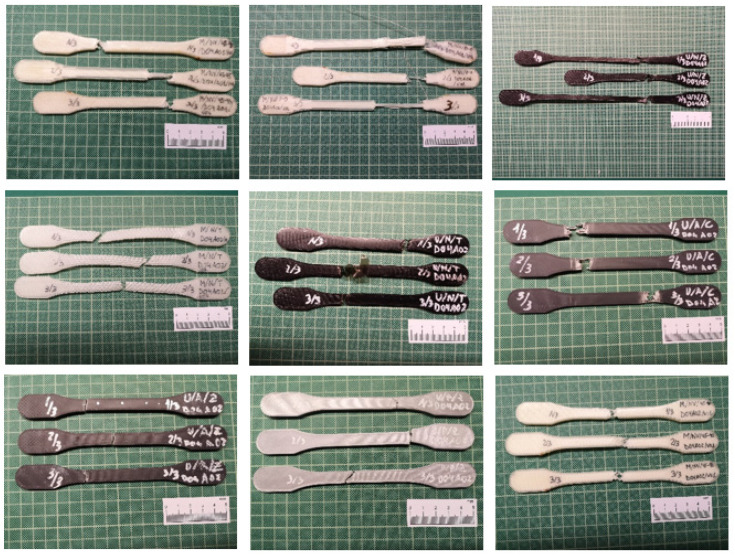
Polymer and composite specimens.

Two 3D machines based on FFF technology were used in this study. Pure polymer specimens were created using the Ultimaker 2 Extended+ printer (Ultimaker, Utrecht, The Netherlands), and composite parts were obtained using the Markforged Mark Two printer (Markforged, Watertown, MA, USA) (Figure 2 and Figure 3).

Table 2 lists the principal technical specifications for these machines. Note that the Ultimaker printer offers a great range of printing parameters, which makes it possible to use a variety of different settings for the layer height, printing speed, infill percentage, wall thickness or extrusion, and printing bed temperature. However, the Mark Two desktop 3D has limited printing possibilities. This restricted the conditions used in this experiment with both printers, making it necessary to focus on these pre-established parameters. 

A nozzle diameter of 0.4 mm and layer height of 0.2 mm were selected for both printers. To avoid the warping effect caused by a decrease in the dimensions of a part during solidification, a 90% infill density was used. Samykano et al. [8] show that the optimum values are reached on a combination of 80% infill fiber, 0.5 mm layer thickness, and 65° raster angle. Elmrabet et al. [40] analyze the 20%, 60%, and 100% infill with different layer thickness options, obtaining the best results with 100% infill in the PLA polymer, at the expense of a deposition temperature increase up to 225 °C. Both the lower and upper paths and the two outer side walls were 100% filled. A compensation retraction parameter was activated to achieve quality prints, and the deposition speed (45 mm/s) recommended by the software specifications was used, which remained constant for the rest of the materials. Furthermore, to facilitate adhesion to the hot build plate, the speed rate was decreased to 15 mm/s for the lower layer. Table 3 lists the printing parameters selected in the Ultimaker printer with the Cura software, based on the materials used for the printed specimens.

Regarding the Mark Two equipment, the Eiger software does not allow substantial modifications of the printing parameters such as the deposition speed and temperature. Thus, it was necessary to use the same parameter configuration for the entire set of specimens.

In order to conduct a reliable comparative evaluation of the two printers, using the nylon material as the link element, the same parameter configuration was used for all the specimens, with the exception of the infill pattern, which was modified to obtain different responses to the applied efforts.

In this study, out of all the infill pattern options provided by the printing software, in both the Ultimaker and Markforged equipment, the triangular option was chosen. For the previously mentioned reason, an infill rate of 90% was used for the printed specimens. Because of the inclusion of reinforcement fibers, specimens were configured with three lower layers, three upper layers, and two side outer walls.

### 2.3. Infill Patterns

Each of the printers had a specific parameter configuration. Therefore, it was necessary to adapt them to obtain similar behaviors. 

The infill pattern of the Ultimaker affected the inner configuration of the polymeric specimen (27 specimens). In this case, a concentric pattern was the most similar to the 0-0 Markforged pattern because the fiber was deposited in a similar in the calibrated zone. This was parallel to the major axis and had the same alignment as the load applied to the sample in the tensile test. The zig-zag pattern was comparable to the 45-90 Markforged model because the deposition started at 45° with respect to the major axis and was then rotated 90° in the next layer with respect to the first one. The triangular pattern was the most isomorphic one, with different deposition directions. Therefore, the sample exhibited a behavior similar to that of an isotropic one. It was necessary to take into account the two outer walls, with were always built with 100% infill and concentric depositions. 

On the other hand, in the Markforged printer, the variation of the deposition pattern only affected the reinforcement fibers, and the same triangular filling pattern was kept for the nylon matrix. 

**Figure 4:**
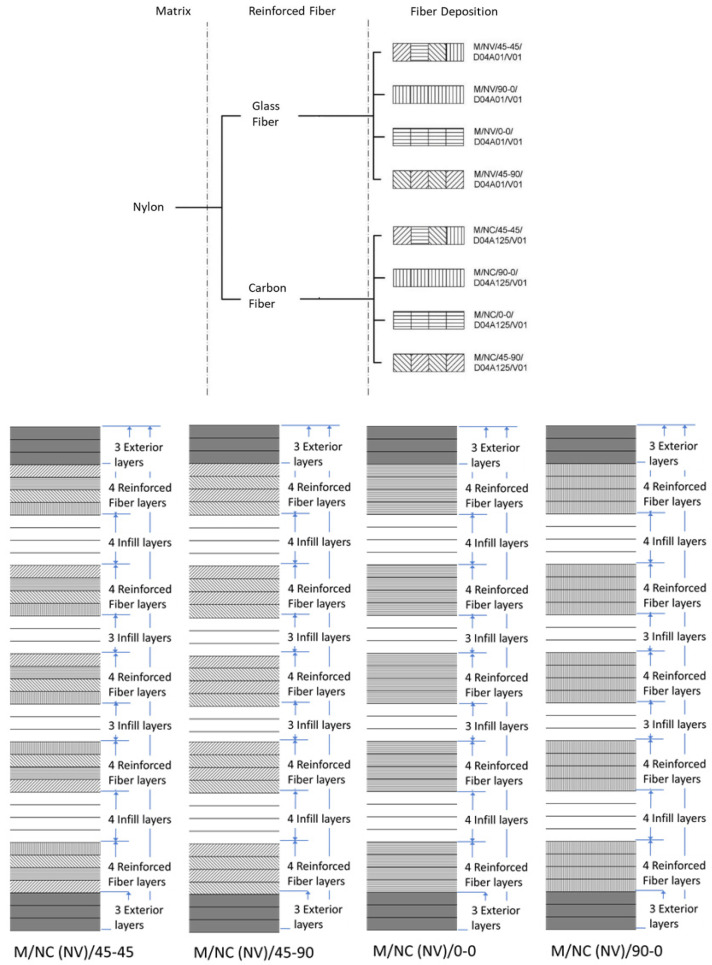
Markforged fiber deposition patterns (above) and layer distribution (below).

To carry out this experiment, 24 specimens were created using four different deposition patterns for both fiberglass and carbon fiber (Figure 4).

The fiber in the sample was distributed in a package form. The upper and lower outer walls were made of nylon layers with a 100% infill density. Next, the first four-layer packet of fiber was incorporated with a defined orientation, and a filling material package was subsequently positioned. This design was repeated, with a symmetrical scheme, until the final thickness of the specimen was reached 24 layers distributed 50% between Nylon and reinforcing fiber (Glass Fiber or Carbon Fiber) layers, which represents a value close to 30% of the fiber–matrix volumes ratio. Pyl et al. [43] work with 47% infill fiber density, ensuring the correct wrapping of the resin around it.

The use of carbon fiber reinforcement implies that the Eiger software used a layer height of 0.125 mm (instead of the 0.1 mm pre-established). Thus, the total number of layers was adjusted to maintain the previously indicated fiber/matrix ratio. The final structure of each type of specimen depended on the fiber material.

Glass fiber and carbon fiber: the sample contained five fiber packets that alternated with the infill material. Each packet had four consecutive reinforcement layers with a variable deposition pattern according to the sample design. 

### 2.4. Sample Design

**Figure 5:**
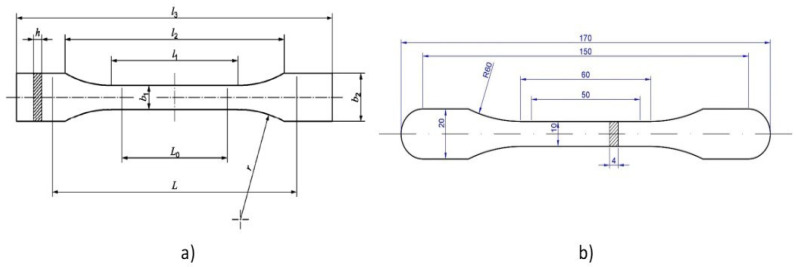
(**a**) Specimen geometry (ISO 527-2) and (**b**) modified specimen geometry.

The geometry of the specimens was designed according to the requirements for determining their tensile properties following the ISO 527-2:2012 standard (Tensile Test for Plastics) (Figure 5a). However, to avoid the samples were removed from the bed during the printing process (due to the warping effect), the end of the grip zone was rounded (Figure 5b). In the end, the 6b specimen with a total length of 150 mm was selected. The significant tapering of the radius between the grip zone and central calibrated zone promoted the occurrence of breakage in the 50 mm long calibrated zone.

### 2.5. Reference Code

The following ad hoc codification was created to identify the specimens (Table 4): Printer/Matrix, Reinforcement/Deposition pattern/Nozzle diameter, Layer height/Version.

As an example, the code M/NV/45-90/D04A01/V01 identifies a specimen manufactured by the Markforged (M) printer, with a nylon (N) matrix and fiberglass as the reinforcement material (V). The numbers 45-90 indicate that the deposition angle of the fiber in the first layer of the packet was 45° with respect to the principal axis and was rotated by 90° for the next layers. D04A02 refers to a nozzle diameter of 0.4 mm and layer height of 0.2 mm. The last part of the code (V01) indicates the current design of the part.

**Figure 6:**
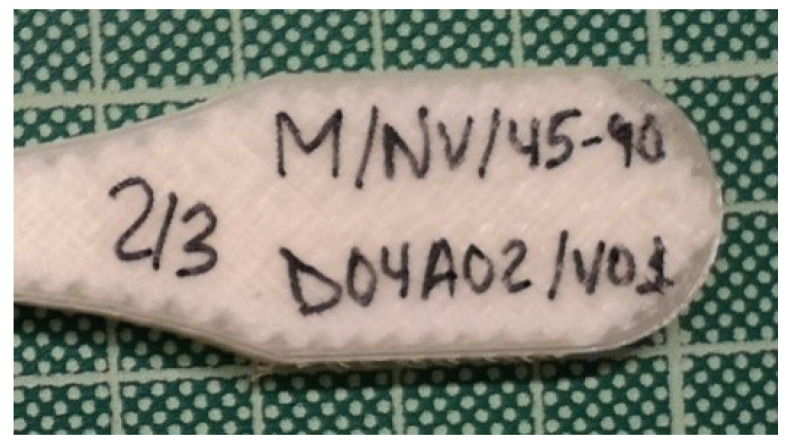
Samples codification.

Once the specimens were printed and distributed based on the same parameter settings, they were numbered and marked on both sides (1/3, 2/3, or 3/3). In this way, they could easily be rejoined after the tensile test was performed (Figure 6).

### 2.6. Tensile Test Machine 

**Figure 7:**
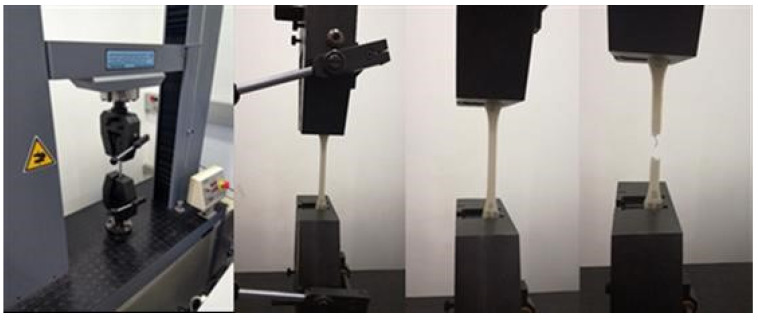
Servosis ME-405 machine and tensile test performance.

The tests were performed on a Servosis ME-405 universal testing machine (Servosis, Madri, Spain), controlled by the PCD2K test software (Servosis). It has a low-force range for the pseudo-static tests of all materials such as plastics and composites under room temperature conditions. Although it is built to handle 1–10 t, the scale was reduced to a 2 t range to obtain greater accuracy. Extensometer has not been used (Figure 7). 

The stress-strain curves (*σ-ε*) have been obtained from the data provided by the test software, determining the tensile stress at yield by fitting the curve to a straight line with offset 0.2% strain.

## 3. Results and Discussion

### 3.1. Results 

The results obtained from the tensile tests performed on the set of specimens made with additive manufacturing using Fused Filament Fabrication are shown in this section. 

Table 5 lists the average values for the main mechanical properties, as determined in the tensile tests, including the tensile modulus, yield strength, tensile strength at break, and elongation. 

Next figures show the evolution of the different parameters according to each deposition patterns and material of this experience (Figures 8–10, 12, 14, 16, 21 and 26) as well as the specific behavior of certain specimens (Figures 11, 13, 17–19 and 23–25).

### 3.2. Discussion

#### 3.2.1. Infill Pattern Influence on PLA Parts Printed Using Ultimaker Printer (U/P/C vs. U/P/Z)

**Figure 8:**
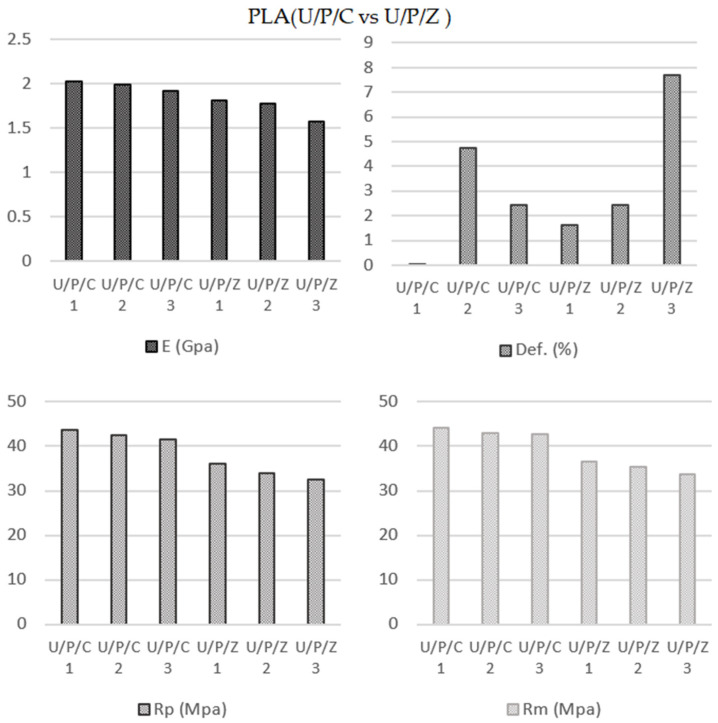
PLA specimens (Concentric vs. Zig-zag) parameters average values.

A comparison of the zig-zag scheme with the concentric one shows decreases of approximately 13.22%, 19.77%, and 18.56% for the modulus of elasticity (1975.67 MPa U/P/C; 1714.00 MPa U/P/Z), yield strength (42.58 Mpa U/P/C; 34.16 MPa U/P/Z), and tensile strength (43.20 MPa U/PC; 35.18 MPa U/P/Z), respectively (Figure 8). However, the percentage of elongation was sensitively higher (slightly more than 63%) (2.41% U/P/C; 3.92% U/P/Z). Zhao et al. [63] obtained 2864.37 MPa for the Yield Modulus and 49.66 MPa for the Tensile Strength with a similar configuration to the 0-0 but using a layer thickness of 0.1 mm (0.2 mm in our case). This way, it is proved that the layer thickness is in inverse proportion to the mechanical properties (Camargo et al. [22]; Aloyaydi et al. [34]). The printed setting defines by concentric deposition pattern (U/P/C) (similar to the 0-0 in the calibrated zone), 0.2 mm layer thickness and y 90% infill, results in higher values of E, Rp and Rm in comparison with the configuration in which the zig-zag deposition pattern is used (U/P/Z).

#### 3.2.2. Infill Pattern Influence on ABS Samples Printed Using Ultimaker Printer (U/A/C vs. U/A/Z)

**Figure 9:**
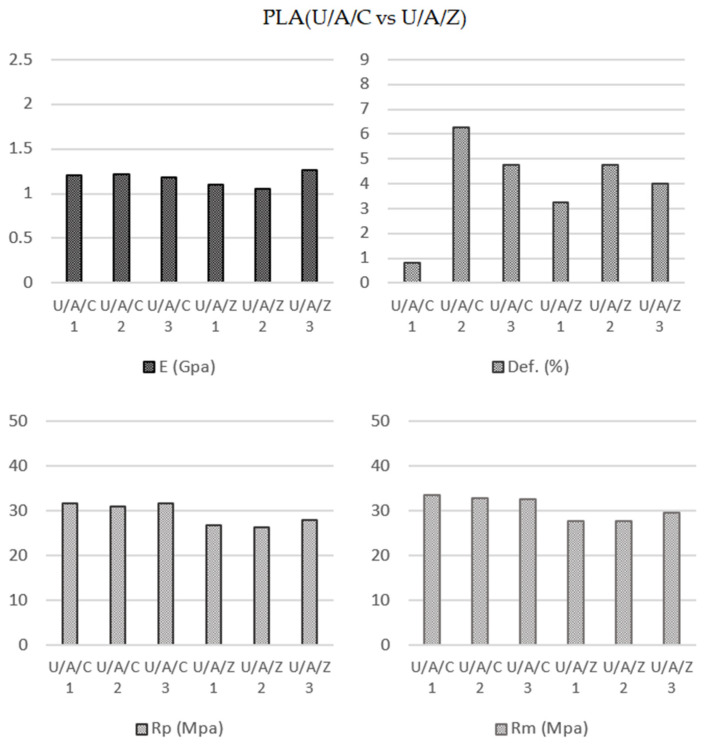
ABS specimens (Concentric vs. Zig-zag) parameters average values.

Following the same method as in the previous case, a comparative evaluation was made of the results obtained by the application of the two types of deposition, concentric, and zig-zag (Figure 9), but, in this case, for ABS material. 

Data provided in Table 5 show that the samples created by the application of the concentric infill pattern had a better mechanical behavior. However, these results were not as relevant as the PLA results. The elasticity modulus values when using the zig-zag pattern were 5.53% smaller than the concentric values (1204.00 MPa U/A/C; 1137 MPa U/A/Z). The yield strength (31.42 MPa U/A/C; 27.04 U/A/Z) and ultimate tensile strength (33.01 MPa U/A/C; 28.38 MPa U/A/Z) had differences of 13.94% and 14.04%, respectively. Referring to the elongation at break (3.95% U/A/C; 4.00% U/A/Z), the samples showed an insignificant increase of 1.26%. Samycano et al. [8], using 80% infill and 0.5 mm layer height, obtained yield modulus of 774.50 MPa; yield strength of 19.95; tensile strength of 31.57 MPa and elongation at break of 9.4%.

#### 3.2.3. Material Influence on Specimens Manufactured by Ultimaker Printer with Concentric Deposition Pattern (U/P/C vs. U/A/C vs. U/N/C)

**Figure 10:**
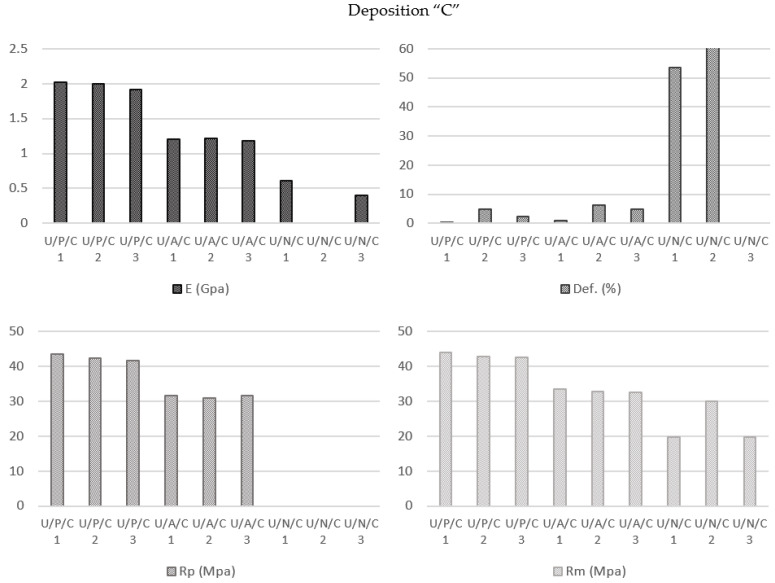
PLA, ABS, and Nylon specimens with Deposition pattern “C” parameters average values.

In this study, a comparative analysis of the results showed the readily foreseeable behaviors of the PLA (Elastic Modulus 1975.67 MPa; Yield Strength 42.58 MPa; Break Strength 43.20 MPa; Elongation at Break 2.41%) and ABS (Elastic Modulus 1204.00 MPa; Yield Strength 31.42 MPa; Break Strength 33.01 MPa; Elongation at Break 3.95%) specimens. However, the response of the nylon samples PLA (Elastic Modulus 505.50 MPa; Break Strength 23.19 MPa; Elongation at Break 57.01%) was clearly different because the concentric infill pattern caused a deformation typical of viscoelastic behavior (Figure 10).

**Figure 11:**
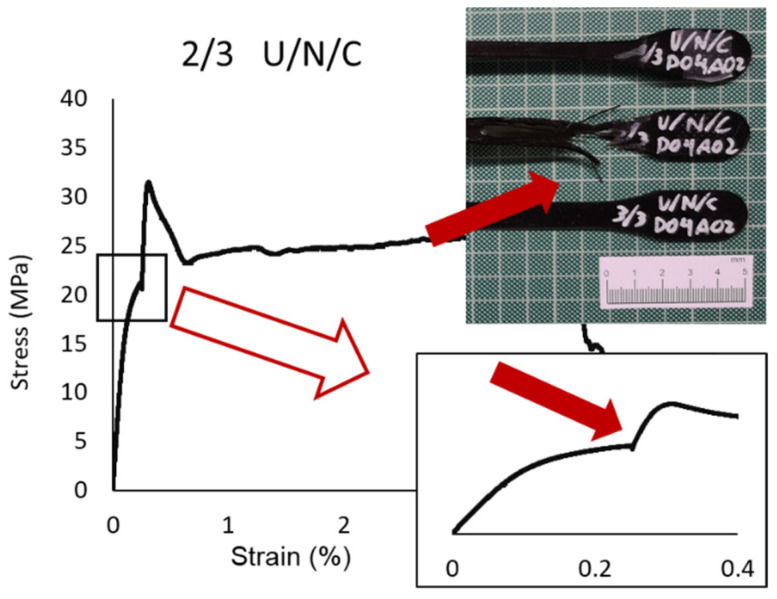
Initial tear on U/N/C sample and its representation on stress-strain graph.

When the deformation was approximately 10%, an initial tear in the shape of a “V” appeared as a result of the fiber structure formed in the zone near the transition radius. From this point, the printing lines became parallel to the load axis, increasing the tensile strength of the material, and finally reaching its definitive rupture point. The initial tear could be considered the effective breakage point of the sample (Figure 11).

#### 3.2.4. Material Influence on Specimens Manufactured by Ultimaker Printer with Zig-Zag Deposition Pattern (U/P/Z vs. U/A/Z vs. U/N/Z)

**Figure 12:**
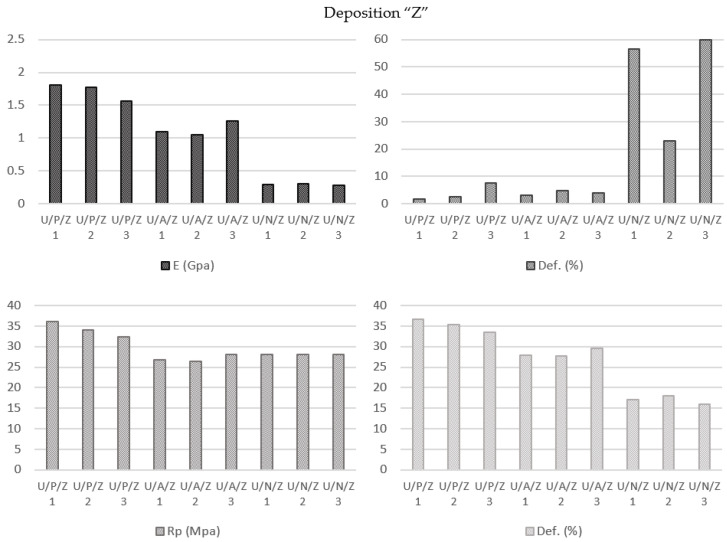
PLA, ABS, and Nylon specimens with Deposition pattern “Z” parameters average values.

For both PLA (Elastic Modulus 1714.00 MPa; Yield Strength 34.16 MPa; Break Strength 35.18 MPa; Elongation at Break 3.92%) and ABS (Elastic Modulus 1137.00 MPa; Yield Strength 27.04 MPa; Break Strength 28.38 MPa; Elongation at Break 4.00%) polymers, this printing configuration followed the same trend as the previous concentric configuration, with decreases in the modulus of elasticity, yield strength, and ultimate tensile strength values of the ABS parts in comparison with the PLA samples (Figure 12) in response to the specific characteristics of each of these materials.

**Figure 13:**
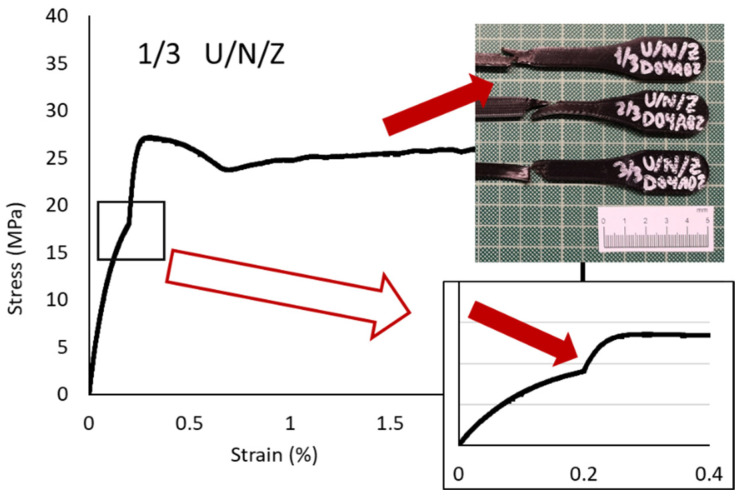
Initial tear on U/N/Z sample and its representation on stress-strain graph.

The behavior of the nylon (Elastic Modulus 291.67 MPa; Yield Strength 28.00 MPa; Break Strength 17.05 MPa; Elongation at Break 46.53%) was also similar to that obtained in the previous analysis, but the “tear” effect was lower because in this zig-zag pattern each and every one of the layers had the same orientation due to the shear strength direction which causes the failure. As shown in (Figure 13), the “V” effect was distorted on the inclined line for the 45° orientation. 

#### 3.2.5. Infill Pattern and Printing Equipment Influences on Nylon Specimens ((U/N/C vs. U/N/Z vs. U/N/T vs. M/N/Z vs. M/N/T)

**Figure 14:**
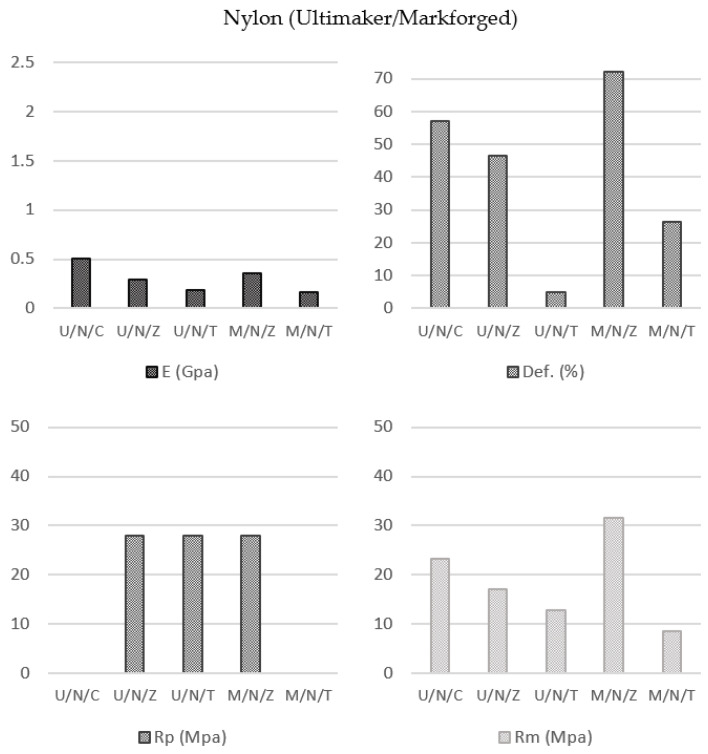
Nylon specimens’ parameters average values.

The goal of this study was to evaluate the influences of the process parameters on the mechanical behaviors of nylon specimens. Therefore, a study batch of specimens were obtained using the specific printing conditions defined in the analysis case and subjected to tensile (Figure 14). 

The results made it possible to observe that the behaviors of the nylon specimens printed using the printers from the two manufacturers were similar as long as the same internal structure was maintained (note the Z and T infill patterns). In fact, the aim of this evaluation is to determine the behavior of the nylon from the Ultimaker printer as compared with the nylon behavior from the Markforged device, as their characteristics are also different. For this reason, the study of the behavior with concentric deposition pattern is not relevant because of not be applied in both printers. 

On the other hand, the T infill pattern (Elastic Modulus: 189.33 MPa U/N/T and 164.67 MPa M/N/T; Yield Strength: 28.00 MPa; Break Strength: 12.73 MPa U/N/T and 8.65 MPa M/N/T) provided worse mechanical properties for the samples than the Z infill pattern (Elastic Modulus: 291.67 MPa U/N/Z and 359.00 MPa M/N/Z; Yield Strength: 28.00 MPa U/N/Z and 28.00 MPa M/N/Z; Break Strength: 17.05 MPa U/N/Z and 31.57 MPa M/N/Z). Moreover, it is remarkable that this format affected the elongation of the material (Elongation at break: 46.53% U/N/Z and 72.04% M/N/Z; 5.01% U/N/T and 26.48% M/N/T).

Comparing both filaments, mechanical properties of the Markforged nylon are better than the ones of the Ultimaker nylon.

These two behaviors, which may seem anomalous in a first analysis, are justified by the fact that in the type of deposition triangular T it is not possible to “package” the filaments. Thus, the filling density is much lower than that presented by other types of patterns.

**Figure 15:**
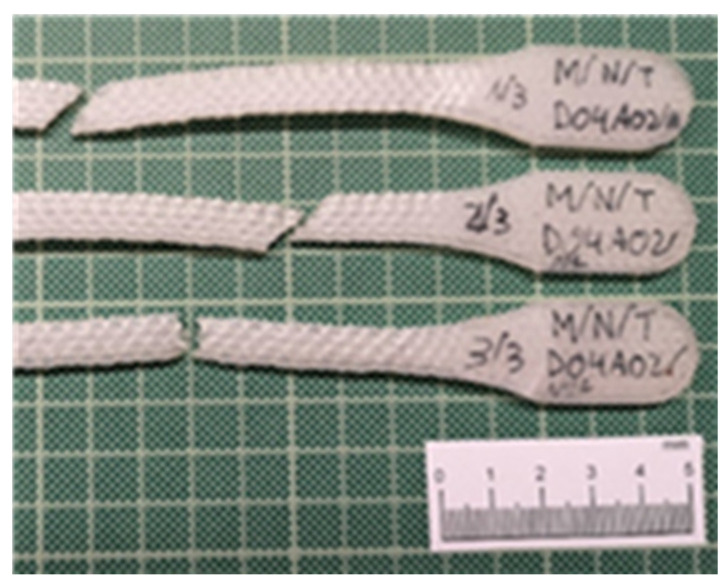
Triangular internal structure of M/N/T specimens.

Finally, the decrease in the elongation percentage was due to the reticulated three-dimensional triangular structure created inside the specimen, which gave it a more effective resistance to deformation (Figure 15).

#### 3.2.6. Infill Pattern Influence on Fiber Type (45-45 vs. 0-0 vs. 45-90 vs. 90-0)

##### Glass Fiber-Reinforced Nylon Polymer ((M/NV/45-45 vs. M/NV/0-0 vs. M/NV/45-90 vs. M/NV/90-0)

**Figure 16:**
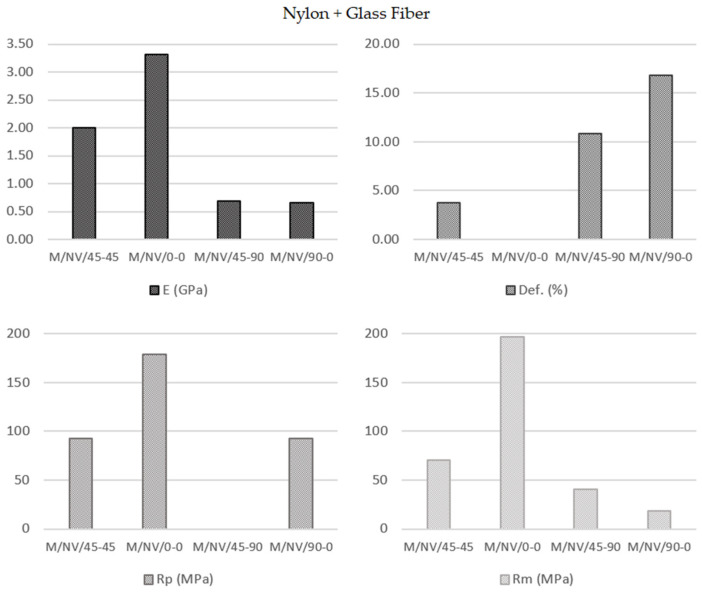
Nylon + Glass fiber specimens’ parameters average values.

At this point, an experiment was conducted on the composite materials, which increased the complexity of the mechanical behavior analysis based on the tensile test results. Initially, it seems that the mechanical property values should be proportional to the nylon matrix and glass reinforcement composition percentages. However, when using the FFF technology, additional variables can significantly modify the expected behavior (Figure 16).

**Figure 17:**
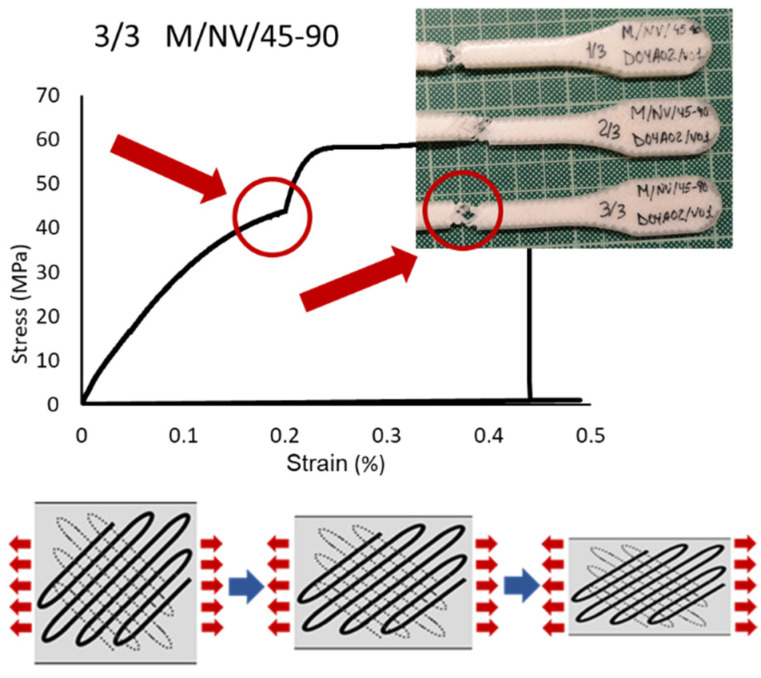
Breakage detail of M/NV/45-90 specimen, stress-strain graph and drawing “X” behavior scheme of the fiber.

**Figure 18:**
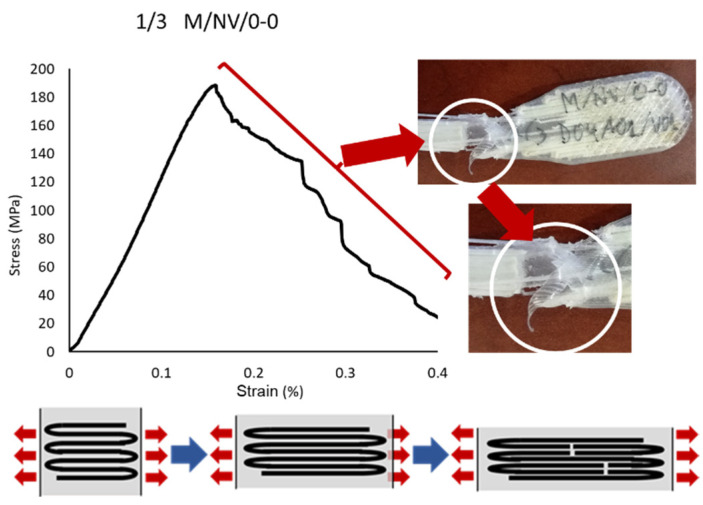
Break detail of 1/3 M/NV/0-0 specimen: stress-strain graph and drawing and breaking behavior scheme of the fiber.

**Figure 19:**
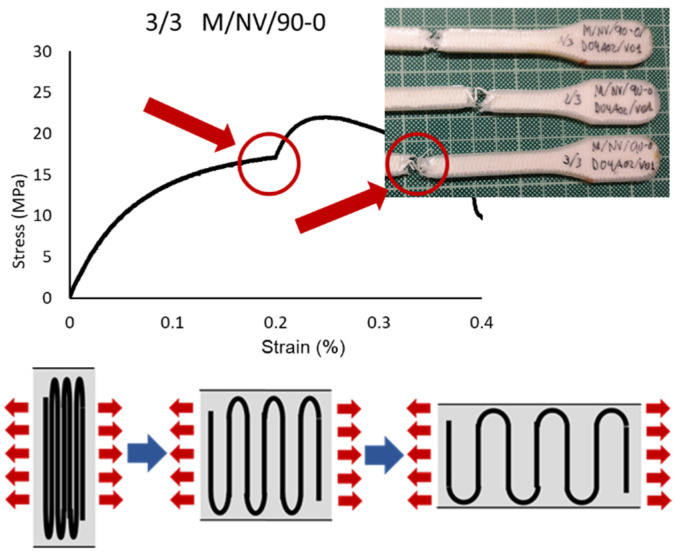
Break detail of 3/3 M/NV/90-0 specimen, stress-strain graph and “spring” behavior of the fiber.

Although initially it seemed that the four types of specimens showed substantially distinct behaviors from each other, it was observed that with different slopes and elastic limits, the specimen were deformed until the ultimate tensile strength was reached. In this sense, the reinforcement fiber had a decisive influence on the mechanical behaviors of the specimens. The particular behavior according to each type of deposition pattern reveals that not only the fiber properties have influence but also its configuration (Figure 17, Figure 18 and Figure 19). 

Concretely, the type 45-90, in which the fiber is arranged in successive layers, with a first layer inclined 45° with respect to the load direction and each one of the next layers rotated 90°, defines a structure in the shape of a “X”. This internal structure is elongated when the resin is given way and broken (identified by the noted point in (Figure 17).

In the 90-0 deposition configuration, a similar case is presented but with significantly lower break strength values (41.04 MPa M/NV/45-90; 18.40 MPa M/NV/90-0) due to the fact that the fiber is separated with a behavior similar to a “spring”, offering no resistance to deformation until resembling 0-0 configuration (Figure 18).

For this reason, the highest deformation value of the M/NV specimens is reached in this type (16.80% M/NV/90-0; 10.87% M/NV/45-90; 3.73% M/NV/45-45; 0.00% M/NV/0-0).

On the opposite, the M/NV/0-0 specimens behave as extremely rigid specimens (0.00% deformation) since the fiber works from the beginning, reaching an exceptional mechanical behavior, with Yield Modulus 3317.00 MPa, Yield Strength 179.18 MPa, and Break Strength 196.72 MPa. Figure 19 shows how, once the Break Strength is reached, the fiber is breaking into small packages, generating the steep line on the graph as a result of the resistance decrease of the specimen.

**Figure 20:**
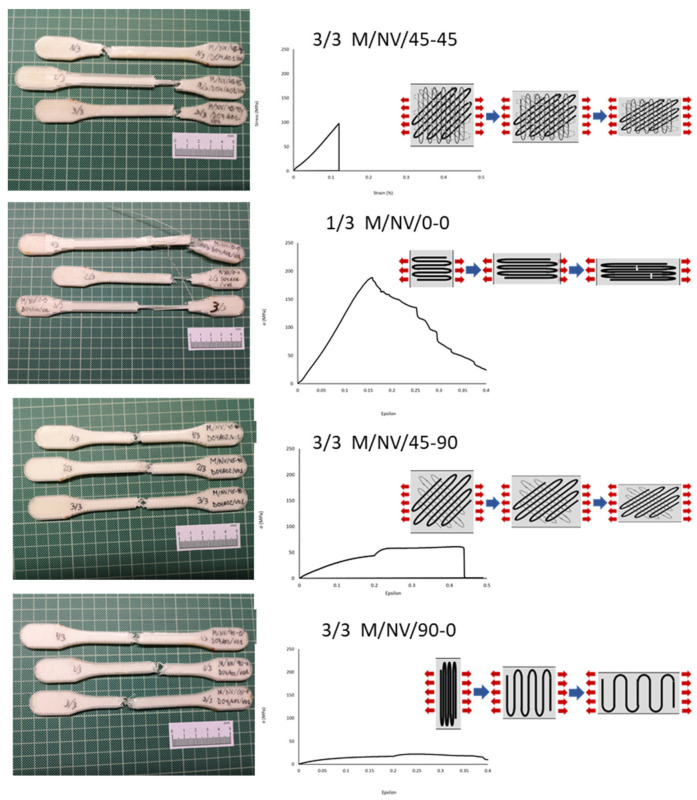
Nylon + Glass Fiber MNV45-45, MNV0-0, MNV45-90, and MNV90-0 specimens; Stress-Strain graphs; and fiber behavior for each deposition pattern.

Figure 20 shows jointly the specimens and the σ–ε graphs of each type of deposition pattern described. The different structures created by means of the overlap between layers in the four different types of specimens clearly showed how the 0-0 orientation, parallel to the direction of the applied load in tensile testing, was the decisive factor of their mechanical strength. For this reason, in the 0-0 configuration, the fiber layers have the same orientation, providing breaking strength values of up to 196.72 MPa for fiberglass and 243.64 MPa with carbon fiber. In the 45-45 design, one in four layers (25%) has a parallel configuration (also a 25% with 90° orientation) what, in combination with the greatest of similarity to an isotropic structure, provided breaking strength values of 70.50 MPa and 108.43 MPa (for NV and NC fibers respectively). The 45-90 configuration neither presents parallel layers to the application of the load (0°) nor perpendicular ones (90°), what makes weak the specimens. Therefore, mechanical strength values are presented in the third place (41.04 MPa for NV and 49.81MPa for NC). Finally, in the 90-0 pattern, the whole fiber layers had a perpendicular orientation to the load direction. Consequently, they are the weakest (18.40 MPa in NV and 27.26 MPa in NC).

The parallel orientation of the fiber offered its maximum strength value, which was decreasing until the 90° orientation in which the fiber weakened and was deforming until reaching the orientation closest to 0°.

To summarize, this analysis showed that orientation of the deposition pattern of the layers has a direct influence on the mechanical behavior of the specimens, which increased depending on the percentage of layers with the 0-0 orientation and decreased according to the number of layers with the 90-0 configuration. (Figure 20 and details in Figure 17, Figure 18 and Figure 19).

##### Carbon Fiber-Reinforced Nylon Polymer (M/NC/45-45 vs. M/NC/0-0 vs. M/NC/45-90 vs. M/NC/90-0)

**Figure 21:**
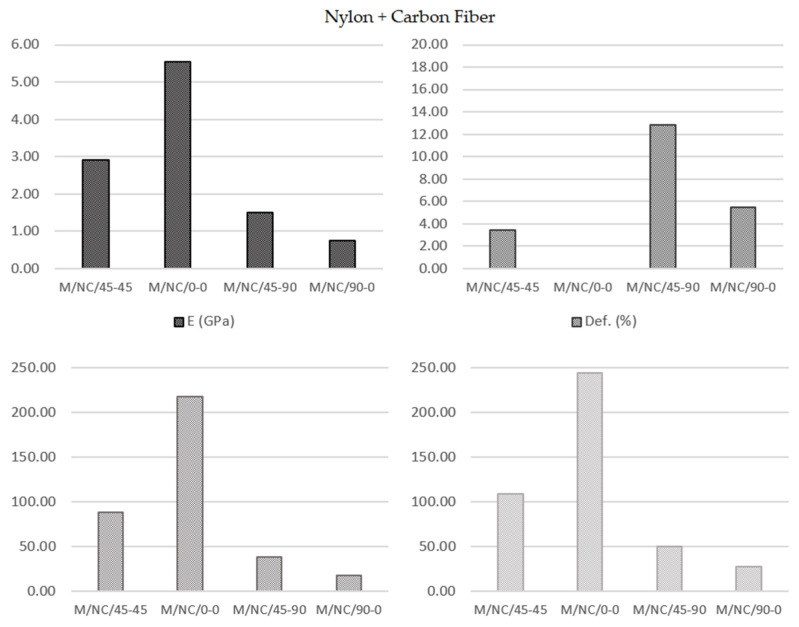
Nylon + Carbon Fiber specimens’ parameters average value.

This analysis was totally analogous to the previous case, with a greater interest from an engineering point of view as the significantly higher values of the mechanical properties. The deformation of the carbon fibers showed a similar evolution, although with lower deformation values (greater stiffness). In contrast, the tensile strength results were much higher (Figure 21).

Blok et al. [31] presented nylon with carbon fiber reinforcement values of Elastic Modulus of 62.5 GPa and Tensile Strength of 986 MPa but with a specific configuration of layers in which the carbon fiber is compactly concentrated. Pyl et al. [43], with 47% fiber volume ratio, showed Elastic Modulus values ranging between 58.07 GPa and 4 GPa and Break Strength values between 719 MPa and 48 MPa, depending, in both cases, on the type fiber deposition.

**Figure 22:**
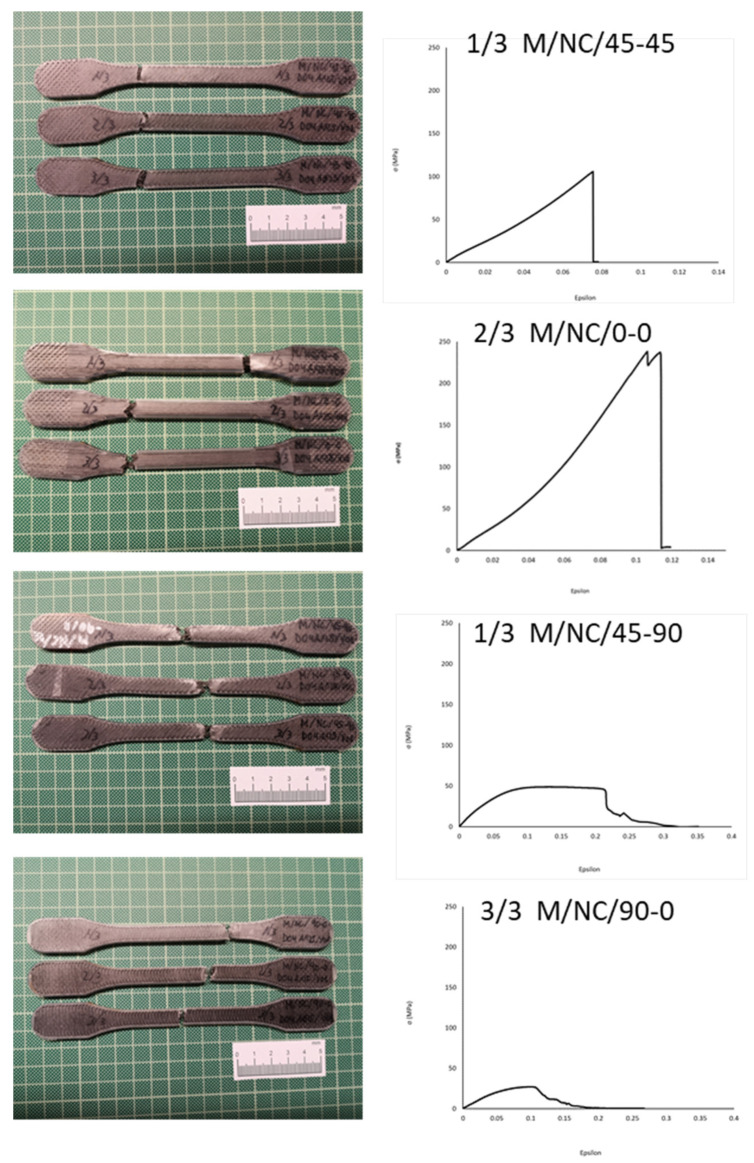
Nylon + CarbonFiber M/NC/45-45, M/NC/0-0, M/NC/45-90, and M/NC/45-90 specimens and Stress-Strain graphs.

**Figure 23:**
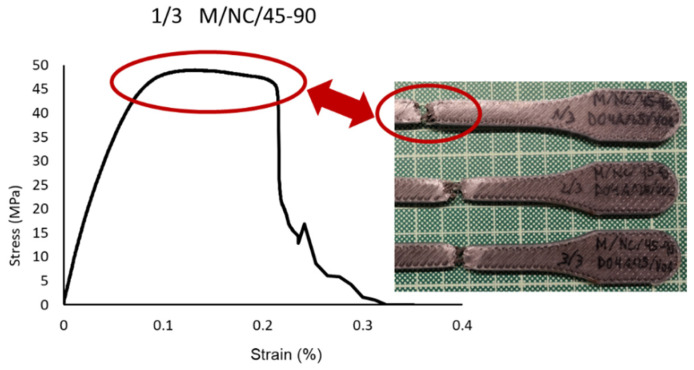
Break detail of 1/3 M/NC/45-90 specimen and stress-strain graph.

**Figure 24:**
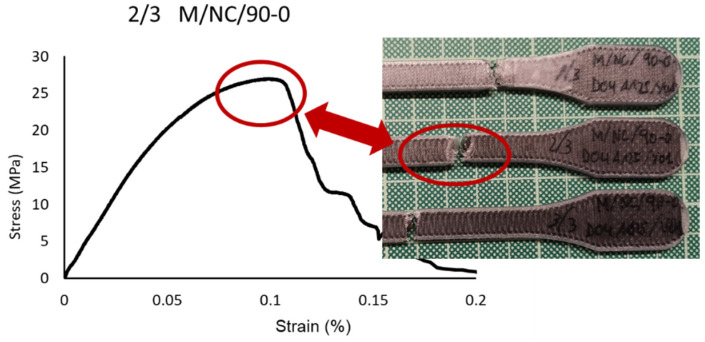
Breakage detail of 1/3 M/NC/90-0 and stress-strain graph.

Figure 22 shows how the evolutions of the four types of samples maintained the same characteristics as the previously studied case. In other words, there was a continuous slope until the nylon matrix broke, after which the fiber packets began breaking. The infill pattern of the fiber acted as a determinant factor for the slope of the curve and therefore the elasticity modulus. It is therefore repeated the effect of the “X” fiber structure in the 45-45 design (Figure 23) and the effect of the “spring” fiber structure in the 90-0 configuration (Figure 24). In this case, the fiber simulated a spring that was uncoiling until situated parallel to the load axis.

**Figure 25:**
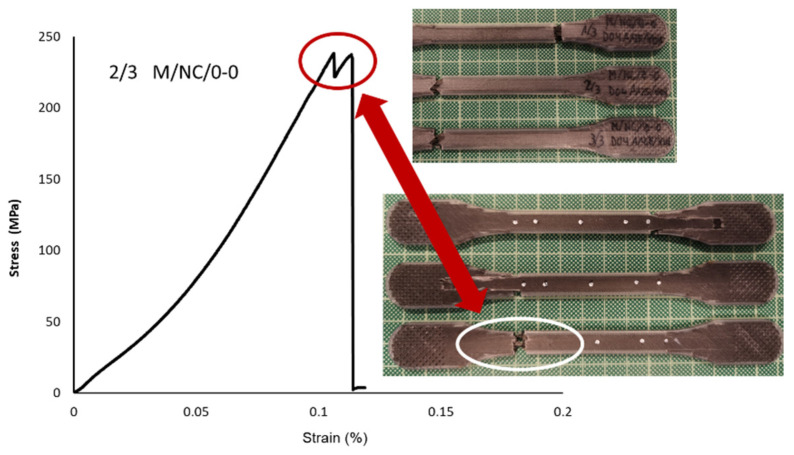
Detail of 2/3 M/NC/0-0 specimen breakage and stress-strain graph.

The 0-0 pattern with fiber orientated parallely to the load applied in the tensile test, the highest value is obtained (Elastic Modulus 554,533 MPa; Yield Strength 217.34 MPa; Tensile Strength 243.64 MPa). The σ–ε curve shown in Figure 25 defines the particular behavior of the specimen with the 0-0 orientation. It was the strongest, with the most brittle failure. The nylon matrix first suffered from delamination, followed by the fiber. The valley highlighted in the curve identifies the instant of rupture.

**Figure 26:**
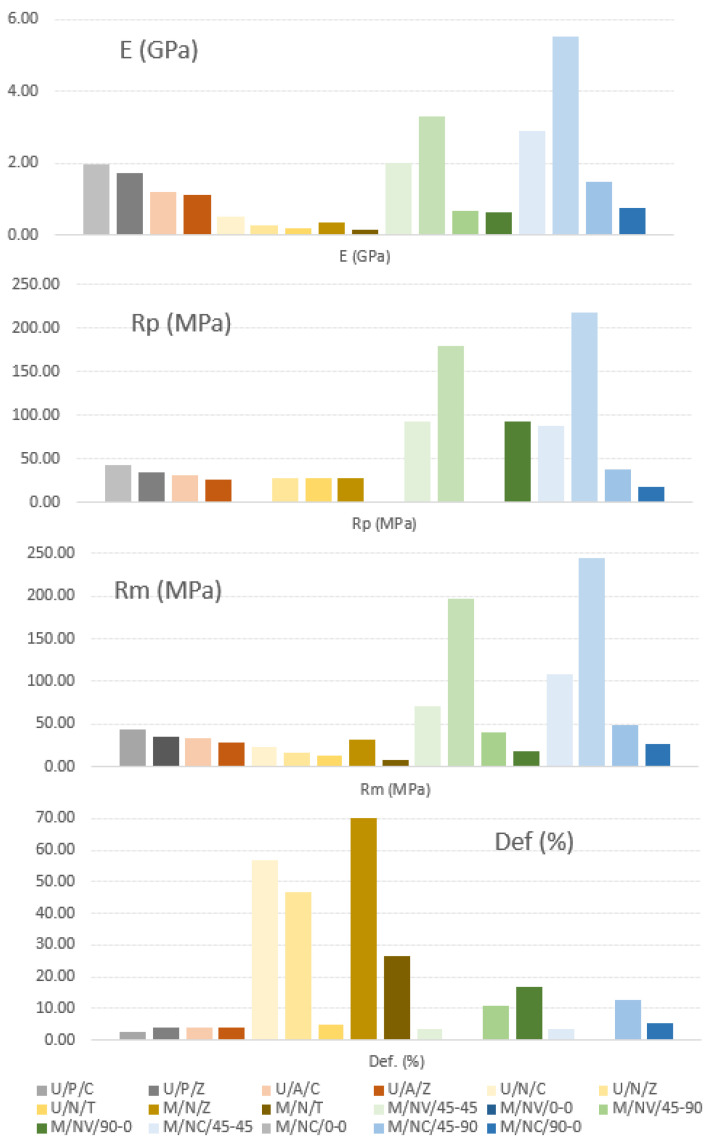
Average E (GPa), Rp (MPa), Rm (MPa), and Def (%) values of PLA/ABS/Nylon specimens.

As a brief overview, the Mean Values of the different parameters taken in account in this project can be seen in the Figure 26.

## 4. Conclusions

This study aims to show the deposition pattern influence on the mechanical behavior of the specimens (polymers and reinforced polymers with carbon or glass fiber) manufactured by an additive manufacturing process (AM) with fused filament fabrication technology (FFF). The parts are built by depositing melted material layer-by-layer, which is intrinsically linked to a decrease in the mechanical properties caused by the imperfect adhesion between layers.

Due to the different characteristics of the two types of specimens studied (polymers and composites), the following conclusions were considered independently.

❖ Deposition pattern influence on polymeric specimens

Using PLA samples, yield modulus values obtained in the tensile test compared to data from the manufacturer showed a decrease by 15.96% in the concentric deposition (U/P/C) and by 27.07% in zig-zag deposition (U/P/Z). The values of the others mechanical parameter were also reduced: tensile stress at yield by 13.98% (U/P/C) and 30.99% (U/P/Z), tensile stress at break by 5.26% (U/P/C) and 22.85% (U/P/Z), and elongation at break by 53.65% (U/P/C) and 24.61% (U/P/Z). The concentric deposition pattern (type C) was more satisfactory in the PLA samples because of their better mechanical response. However, they showed a clear decrease in deformation, due to a slight stiffness.

For the ABS specimens, the differences between the data provided by the manufacturer and those obtained from the tensile test have meant for tensile modulus a decrease by 28.33% for concentric deposition (U/A/C) and 32.32% in zigzag design (U/A/Z). This decrease takes values for tensile stress at yield of 19.43% (U/A/C) and 30.66% (U/A/Z), tensile stress at break 2.62% (U/A/C) and 16.28% (U/A/Z), and elongation at break 17.71% (U/A/C) and 16.67% (U/A/Z).

Similar to the use of PLA, in ABS samples, the concentric deposition pattern presented the greatest interest. In addition, in this case the elongation at break was also kept at adequate values, according to the characteristics of the process.

In Nylon specimens (Ultimaker, Markforged), three types of deposition patterns were established, obtaining the following results in terms of variation in comparison with data provided by the manufacturer: tensile modulus decrease by 12.84% (U/N/C), 49.71% (U/N/Z) and 67.35% with triangular deposition (U/N/T); 78.88% (M/N/Z) and 90.31% (M/N/T). Tensile stress at yield was reduced by 0.71% (U/N/Z), 0.71% (U/N/T) and 0.71% (M/N/Z). The decrease for tensile stress at break was 32.59% (U/N/C), 50.43% (U/N/Z), 63.00% (U/N/T), 12.31% (M/N/Z) and 75.97% (M/N/T). Finally, the elongation at break was reduce by 72.85% (U/N/C), 77.84% (U/N/Z), 97.61% (U/N/T), 51.97% (M/N/Z) and 82.34% (M/N/T).

Following the previous cases, findings showed the concentric deposition as the pattern with a lower reduction over the initial values. It remarkable the fact that the triangular design induced a notable stiffness of the specimens and consequently reducing their elongation percentage.

As a result, taking in account the restrictions of this study, the concentric deposition is considered the best pattern in the manufacturing of polymeric parts (PLA, ABS, Nylon)

❖ Deposition pattern influence on reinforced polymer specimens.

Adding reinforcement fiber to the initial polymeric specimens produced a dramatic change on their mechanical behavior, significantly improving the parameters under study.

Both composite specimens (carbon and glass fiber reinforcement) were obtained by means of four fiber deposition configurations, following the orientation of each layer (45-45; 0-0; 45-90; 90-0).

In relation to the nylon specimens studied previously (with different deposition patterns), in nylon reinforced with glass fiber (NV) specimens an increase range of the tensile modulus value, from the 30.36% using the 90-0 configuration up to 556% with the 0-0 deposition, was observed. The tensile strength at yield increase fluctuated from 232% (90-0) to 540% (0-0). For tensile strength at break, a decrease by 41.72% in type 90-0 was produced, but with 0-0 configuration the increase was by 523%. The 45-45 and 45-90 configurations showed results in the middle of both limit values.

As a main conclusion, the 0-0 orientation (fibers oriented in the same direction that the load application) resulted the suitable solution to maintain the high values of the parameters considered.

In parts subjected to loads applied in different directions, the 45-90 orientation and mainly the 45-45 one, are confirmed as the most satisfactory pattern because of their isotropic behavior.

The behavior of the nylon specimens with carbon fiber reinforcement (NC) is completely similar to that exposed in relation to the glass fiber but additionally presenting increased values due to the specific characteristics of the carbon fiber. The absolute maximum values in the three parameters studied in this analysis were shown in this type of samples. Such increase variations are tensile modulus 996% in 0-0 configuration and 46.78% in 90-0, tensile strength at yield 676% in 0-0 and a decrease by 36.93% in 90-0, and tensile strength at break 671% in 0-0 orientation and a decrease by 13.65% in 90-0.

The 45-45 and 45-90 configurations offered intermediate values between the limits considered (0-0 and 90-0).

As in the previous analysis, the fiber with the 0-0 orientation is demonstrated as the most suitable option, in terms of the mechanical behavior of the specimens.

An additional consideration regarding deformations, both for specimens with fiber glass reinforcement and for those with carbon fiber in any of the deposition orientations studied, is the fact that the deformations do not respond to the best pattern determined previously (values optimal for 0-0) as in each type of deposition the fiber adopts a different behavior, participating in the resistance to the load to which the specimen is subjected with different level and different evolution (“X” structure, “spring” effect, etc.) already studied in previous sections.

## Figures and Tables

**Table 1:** Mechanical tensile properties of materials (data from supplier, carbon and glass fiber tests (ASTM) D3039).

Equipment	Material	Tensile Modulus (GPa)	Tensile Stress at Yield (MPa)	Tensile Stress at Break (MPa)	Deformation (%)
Ultimaker	PLA	2.35	49.5	45.6	5.2
Ultimaker	ABS	1.68	39	33.9	4.8
Ultimaker	Nylon	0.58	27.8	34.4	210
Markforged	Nylon	1.70	51	36	150
Markforged	Glass Fiber	21	---	590	3.8
Markforged	Carbon Fiber	60	---	800	1.5

**Table 2:** Ultimaker and Markforged technical specifications.

	Ultimaker 2 Extended +	Markforged Mark Two
Minimum layer height	20 μm (0.02 mm)	100 μm (0.1 mm)
Printing volume	223 mm × 223 mm × 305 mm	320 mm × 132 mm × 154 mm
Materials	PLA, ABS, Nylon	Nylon, Carbon Fiber, Glass Fiber
Technology	FFF	FFF
Coordinate system	Cartesian	Cartesian
Software	Cura	Eiger
Control of parameters	Yes	No
Advantages	Low surface roughness	High mechanical properties

**Table 3:** Ultimaker/Cura printing parameters.

Parameters	ABS/PLA/Nylon	Parameters	ABS/PLA/Nylon
Nozzle diameter (mm)	0.4	Adherence	No
Layer height (mm)	0.2	Thickness wall (mm)	0.75
Infill rate (%)	90	N°. lines/wall	3
Infill pattern	Concentric, Zig-zag	N°. lower and upper layers	3
Temp. extruder (°C)	250/210/250	Sup/Inf. Layers pattern	Linear
Temp. plate (°C)	80	Velocity (mm/s)	45
Support material	No	First layer speed (mm/s)	15

**Table 4:** Reference code.

	Codification
Printer	Ultimaker (U); Markforged (M)
Matrix	PLA (P); ABS (A); Nylon (N)
Reinforcement (Nylon matrix)	Glass Fiber (V); Carbon Fiber (C)
Deposition pattern	Concentric (C); Zig-zag (Z); Triangular (T)
Nozzle diameter	D04 (0.4 mm)
Layer height	A02 (0.2 mm)
Version	V01

**Table 5:** Average values from tensile test results.

		Tensile Modulus (MPa)	Tensile Stress at Yield (MPa)	Tensile Stress at Break (MPa)	Def. 50 mm (%)
Ultimaker 2 Extended+	U/P/C	1975.67	42.58	43.2	2.41
	U/P/Z	1714	34.16	35.18	3.92
	U/A/C	1204	31.42	33.01	3.95
	U/A/Z	1137	27.04	28.38	4
	U/N/C	505.5	---	23.19	57.01
	U/N/Z	291.67	28	17.05	46.53
	U/N/T	189.33	28	12.73	5.01
	M/N/Z	359	28	31.57	72.04
Markforged Mark Two	M/N/T	164.67	---	8.65	26.48
	M/NV/45-45	1995.67	92.97	70.5	3.73
	M/NV/0-0	3317	179.18	196.72	0
	M/NV/45-90	684.33	---	41.04	10.87
	M/NV/90-0	659	92.97	18.4	16.8
	M/NC/45-45	2913.33	88.09	108.43	3.47
	M/NC/0-0	5545.33	217.34	243.64	0
	M/NC/45-90	1490.33	37.92	49.81	12.82
	M/NC/90-0	742.33	17.66	27.26	5.51

## Data Availability

Not applicable.
